# Subclinical Oxidative and Matrix-Regulatory Alterations Associated with Cigarette Smoking and E-Cigarette Use in Periodontally Healthy Adults: A Cross-Sectional Study

**DOI:** 10.3390/jcm15031026

**Published:** 2026-01-27

**Authors:** Fatma Soysal, Fatma Oner, Zeliha Guney, M. Sepehr Zarinkamar, Kamyar Shahsavani, Muhittin A. Serdar, Ceren Gokmenoglu

**Affiliations:** 1 Department of Periodontology, Faculty of Dentistry, Ankara Medipol University, 06570 Cankaya, Türkiye; 2 Department of Periodontology, Faculty of Dentistry, Istinye University, 34010 Istanbul, Türkiye; 3 Faculty of Dentistry, Ankara Medipol University, 06570 Cankaya, Türkiye; 4 Department of Medical Biochemistry, School of Medicine, Acıbadem University, 34750 Istanbul, Türkiye

**Keywords:** electronic nicotine delivery systems, cigarette smoking, oxidative stress, forkhead box protein O1, matrix metalloproteinase 9

## Abstract

**Background/Objectives**: Cigarette smoking is a well-established risk factor for periodontal tissue damage caused by oxidative stress and increased proteolytic activity. Electronic cigarettes (e-cigarettes), marketed as less harmful alternatives, deliver nicotine and reactive compounds that may similarly disrupt periodontal health. However, their molecular effects on clinically healthy periodontal tissues remain unclear. This study aimed to compare oxidative stress-related and matrix-degradative biomarkers in the gingival crevicular fluid (GCF) of cigarette smokers (CS), e-cigarette (EC) users, and non-smokers (NS), and to examine the relationships among these markers. **Methods**: Sixty individuals, who were systemically and periodontally healthy (20 CS, 20 EC, and 20 NS), were examined. Clinical parameters, including probing depth (PD), clinical attachment level (CAL), plaque index (PI), and bleeding on probing (BOP), were recorded. GCF samples were analyzed for reactive oxygen species (ROS), matrix metalloproteinase-9 (MMP-9), and forkhead box protein O-1 (FOXO-1) using ELISA. Initial group comparisons were descriptive, followed by analysis of covariance (ANCOVA) to adjust for age; PI and PD were included as covariates in separate models. Correlations were assessed using Spearman’s analysis. **Results**: PD was significantly higher in both EC users and CS compared with NS (*p* = 0.022). MMP-9 levels were significantly higher in CS than in EC users and NS (*p* < 0.05), while FOXO-1 concentrations were significantly lower in CS compared with NS (*p* = 0.0227). ROS levels did not differ significantly among groups (*p* > 0.05). After adjustment for age, PI, or PD, group differences in MMP-9 and FOXO-1 remained statistically significant, whereas ROS levels remained comparable. FOXO-1 demonstrated positive correlations with ROS and MMP-9 within exposure groups; these associations were considered exploratory. **Conclusions**: In this cross-sectional study, CS and EC use were associated with altered matrix-regulatory biomarker profiles in clinically healthy periodontal tissues, independent of age and periodontal indices. Causal or temporal inferences cannot be drawn, and longitudinal studies are needed to clarify the long-term periodontal implications of these findings.

## 1. Introduction

Periodontal diseases are chronic inflammatory conditions characterized by the progressive destruction of the tooth-supporting tissues. Their pathogenesis involves complex interactions between microbial biofilms and the host immune–inflammatory response, further influenced by environmental and behavioral factors [1,2]. Among these factors, tobacco smoking is a well-established risk determinant that contributes to greater attachment loss, alveolar bone resorption, and impaired healing capacity in periodontal tissues [3,4]. Clinically, smokers exhibit deeper probing depths, greater clinical attachment loss, and elevated levels of pro-inflammatory mediators, including interleukin-1beta (IL-1β), IL-6, tumor necrosis factor-alpha (TNF-α), and matrix metalloproteinase-8 (MMP-8), in gingival crevicular fluid (GCF), reflecting an enhanced inflammatory and oxidative stress response [4,5]. Importantly, molecular alterations in periodontal tissues can occur before clinical signs appear and may increase tissue vulnerability, even in individuals who seem healthy.

Electronic cigarettes (e-cigarettes) have recently emerged as alternative nicotine delivery systems and are often perceived as a ‘less harmful’ alternative to conventional cigarettes. E-cigarettes (EC) are handheld, battery-powered devices that generate aerosol by heating an e-liquid composed of propylene glycol or vegetable glycerin, flavoring agents, and varying nicotine concentrations [6,7]. The physicochemical and toxicological properties of the resulting aerosol depend on the composition of the e-liquid and the heating temperature [8]. Although EC eliminates tobacco combustion, they continue to deliver biologically active nicotine and other reactive compounds that interact with oral tissues [9]. Experimental and clinical studies demonstrate that EC aerosols contain reactive oxygen species (ROS), aldehydes, and trace metals, which can induce oxidative stress, DNA damage, and apoptosis in oral epithelial and periodontal ligament cells [9,10]. However, the translation of these findings to clinically healthy periodontal tissues remains uncertain.

Clinical evidence regarding the periodontal effects of EC use is emerging and is less consistent than that for conventional smoking. Some studies suggest that EC users may exhibit fewer overt inflammatory signs than cigarette smokers (CS); nevertheless, recent reports indicate potential associations with altered subgingival microbial profiles, changes in GCF inflammatory mediators, and less favorable periodontal treatment outcomes compared with non-smokers (NS) [11,12,13]. Notably, most available data focus on individuals with existing periodontal disease, and the biological effects of EC exposure in periodontally healthy individuals remain largely unexplored.

Oxidative stress refers to an imbalance between the production of ROS and the body’s capacity to neutralize them or repair resulting damage. This imbalance directly links nicotine exposure to periodontal tissue destruction [14,15]. Forkhead-box protein O-1 (FOXO-1) serves as a central transcriptional regulator of the oxidative stress response. It regulates redox homeostasis, apoptosis, and inflammatory gene expression while promoting antioxidant defenses [16,17]. Elevated oxidative stress activates FOXO-1, which may subsequently upregulate matrix metalloproteinase-9 (MMP-9), contributing to extracellular matrix breakdown and facilitating cell migration and tissue remodeling under physiological conditions. However, sustained MMP-9 activity accelerates periodontal connective tissue destruction and alveolar bone resorption [18,19,20]. ROS thus acts as both cytotoxic agents and signaling molecules in tissue destruction, and cigarette smoking has been shown to increase ROS production and enhance MMP-mediated matrix degradation [14]. While cigarette smoking has been shown to enhance ROS generation and MMP-mediated matrix degradation, the extent to which EC exposure influences FOXO-1-related pathways in healthy periodontal tissues remains poorly characterized.

Despite their rapidly growing popularity, the impact of EC on healthy periodontal tissues remains largely unexplored, leaving a critical gap in our understanding of their actual biological consequences. Given the limited and evolving evidence, the present study was designed to explore whether EC use is associated with alterations in oxidative stress-related and matrix-regulatory biomarkers in clinically healthy periodontal tissues. We hypothesized that EC users may exhibit subclinical biochemical changes that partially overlap with those observed in conventional CS. Accordingly, this study aimed to evaluate clinical periodontal parameters and key oxidative and matrix-related biomarkers in GCF and to compare findings among EC users, CS, NS.

## 2. Materials and Methods

### 2.1. Study Design

**Figure 1:**
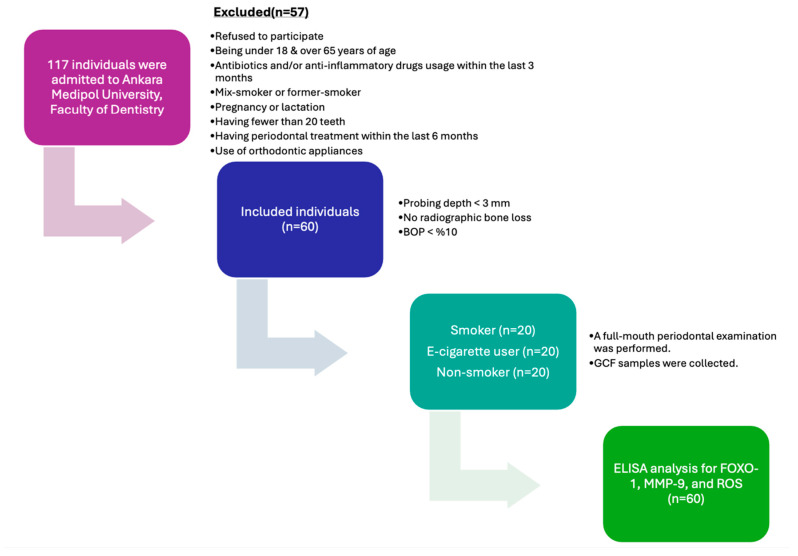
Flow chart and study protocol (BOP: bleeding on probing; GCF: gingival crevicular fluid; ROS: reactive oxygen species; MMP-9: matrix metalloproteinase-9; FOXO-1: forkhead box protein O-1).

This cross-sectional study was approved by the Human Research Ethics Committee of Ankara Medipol University (Approval No: 80, dated 16 April 2025) and was conducted in full compliance with the Declaration of Helsinki. All participants provided written informed consent prior to inclusion. The study was carried out at the Department of Periodontology, Faculty of Dentistry, Ankara Medipol University. A total of 60 systemically and periodontally healthy individuals, including 20 cigarette smokers (CS, *n* = 20), 20 e-cigarette users (EC users, *n* = 20), and 20 non-smokers (NS, *n* = 20), were included in the study. A total of 117 individuals were examined for this study. A total of 21 of the 117 individuals declined to participate in the trial. Four patients were eliminated because they had recently taken antibiotics, nine patients were discarded because they had recently received periodontal therapy, three were excluded because they had systemic diseases, and nine were excluded because they had a mixed-smoking habit, and eleven were excluded because they were former smokers, resulting in a final sample size of 60 systemically and periodontally healthy participants, as presented in Figure 1. Baseline demographic and periodontal variables were compared across groups to identify potential imbalances.

### 2.2. Smoking Status

Smoking status was defined according to the Centers for Disease Control and Prevention (CDC) criteria. Current cigarette smokers were individuals who had smoked at least 100 cigarettes in their lifetime and who currently smoked daily or occasionally. Never-smokers were those who had never smoked or had smoked fewer than 100 cigarettes in total. Smoking habits were assessed using a structured questionnaire, recording the duration of cigarette smoking (years) and the average number of cigarettes smoked per day. Participants in the EC group reported exclusive EC use for at least two years. For these individuals, exposure characteristics included the duration of EC use (years) and the average number of puffs per day.

### 2.3. Clinical Examination

All clinical assessments were performed by a single calibrated examiner (F.S). Prior to participant enrollment, examiner calibration was conducted to ensure measurement reliability. Calibration involved repeated measurements of probing depth (PD) and clinical attachment level (CAL) at six sites per tooth in ten randomly selected individuals on two separate occasions, one week apart. Intra-examiner reliability for continuous periodontal measurements was assessed using the intraclass correlation coefficient (ICC), which demonstrated good-to-excellent agreement (ICC = 0.86 for PD and ICC = 0.88 for CAL). Intra-examiner reliability for bleeding on probing (BOP), a dichotomous variable, was evaluated using Cohen’s kappa coefficient, indicating substantial agreement (κ = 0.82).

Periodontal parameters, including plaque index (PI), probing pocket depth (PD), clinical attachment level (CAL), and bleeding on probing (BOP), were recorded at six sites per tooth (mesiobuccal, midbuccal, distobuccal, mesiolingual, midlingual, and distolingual) using a standardized periodontal probe (UNC 15 Color-Coded Probe, Hu-Friedy, Chicago, IL, USA). Diagnostic criteria were based on the 2017 World Workshop classification [21]. Participants with no clinical or radiographic evidence of alveolar bone loss and a full-mouth bleeding score < 10% were classified as periodontally healthy.

### 2.4. GCF Sampling

GCF samples were collected from standardized sites in the maxillary incisor region to minimize site-to-site variability and improve comparability between participants. Specifically, GCF was obtained from three maxillary single-rooted incisors per participant, selected based on the absence of clinical signs of inflammation. The maxillary incisor region was chosen due to its accessibility, relatively uniform anatomy, and lower susceptibility to plaque accumulation and saliva contamination compared with posterior teeth.

Prior to sampling, the area was isolated with cotton rolls; supragingival plaque, if present, was gently removed with sterile curettes; and the tooth surfaces were air-dried. GCF samples were collected using the extra-sulcular method [22]. Standardized paper strips (Periopaper, ProFlow) were sequentially inserted into the gingival crevice for 30 s at each site. Two strips per tooth were collected and pooled per participant for subsequent analysis. Samples contaminated with blood were excluded. 

GCF volume was quantified using a calibrated electronic device (Periotron 8000, Oraflow Inc., Lynbrook, NY, USA), and instrument readings were converted to microliters using a standard calibration curve [23]. Samples were stored at −80 °C until biochemical analysis.

### 2.5. Quantification of FOXO-1, MMP-9, and ROS

FOXO-1, MMP-9, and ROS concentrations in GCF samples were measured using commercially available enzyme-linked immunosorbent assay (ELISA) (BT LAB, Korain Biotech, Shanghai, China, # E0628Hu and # E0936Hu, for FOXO-1 and MMP-9, respectively; Shanghai YL Bioteck, Co., Ltd., Shanghai, China, # YLA2047HU for ROS), according to manufacturers’ protocols. Absorbance readings were taken at 450 nm using a microplate reader. Concentrations were calculated according to standard curves generated for each assay. Given the short half-life and high reactivity of individual ROS molecules, this method does not quantify free radicals directly but rather detects stable ROS-related oxidation products that serve as indirect indicators of local oxidative stress. Accordingly, the ROS values obtained in this study should be interpreted as a proxy for oxidative burden within the gingival microenvironment.

All samples were analyzed in duplicate, and the mean values were used for statistical evaluation. The intra- and inter-assay coefficients of variation were <10% for all assays. The assay sensitivities were 0.024 ng/mL for FOXO-1, 15.12 ng/mL for MMP-9, and 2.01 U/L for ROS, with detection ranges of 0.05–20 ng/mL, 30–9000 ng/L, and 5–4000 U/L, respectively. 

For quantitative standardization, the measured concentrations were adjusted according to the volume of GCF collected at each site. Final results were expressed as the total amount of each biomarker at each sampling site, enabling site-specific comparisons among groups.

### 2.6. Power and Statistical Analysis

The sample size was calculated using the GraphPad Prism version 10.0 (GraphPad Software, San Diego, CA, USA). Initially, the calculation was based on MMP-9 levels in GCF, defined as the primary outcome variable, following the study by Ye et al. [24]. Although the referenced study quantified MMP-9 using a multiplex bead-based immunoassay rather than an ELISA-based platform, both techniques are antibody-based sandwich immunoassays applied to the same biological matrix (GCF) and within a relevant exposure context. Mean ± standard deviation values reported for the three groups (95.09 ± 50.41, 48.08 ± 35.41, and 38.07 ± 26.67) yielded an estimated effect size of 0.493. Assuming a statistical power of 80% and a significance level of 5% (α = 0.05), the required sample size was calculated to be 15 participants per group. 

To further support the adequacy of the sample size, additional sensitivity calculations were performed using other relevant biochemical and clinical outcome variables reported in the literature, including FOXO-1 concentrations, total oxidant capacity as a surrogate for reactive oxygen species (ROS), and bleeding on probing (BOP). For FOXO-1 concentrations as a secondary outcome variable, the reported [25] mean and standard deviation values (0.446 ± 0.234 for the test group and 0.241 ± 0.114 for the control group) yielded a standardized effect size of −1.114. With a two-tailed significance level of α = 0.05 and a desired statistical power of approximately 0.80, the minimum required sample size was 14 participants per group. 

Because no clinical study has directly evaluated ROS levels, total oxidant capacity (TOC) was used as a surrogate biomarker for ROS in the sample size analysis. Based on reference data [26], following surgical periodontal therapy (889.1 ± 134.85 for the test group and 531.88 ± 78.24 for the control group), as reported by Lütfioğlu et al., the standardized effect size was calculated as −3.24. With α = 0.05 and 80% power, the minimum required sample size was estimated at four participants per group.

In addition, a sample size calculation was conducted, with bleeding on probing (BOP) as the clinical outcome variable. Mean ± standard deviation values reported in a prior study by Ibraheem et al. [27] as 15.4 ± 2.5 for CS, 14.5 ± 0.8 for EC users, and 21.5 ± 3.3 for NS, yielded a standardized effect size of 0.942, corresponding to a minimum sample size requirement of five participants per group at α = 0.05 and 80% power.

Given the largest sample size requirement among all outcome variables and accounting for potential data loss during sampling and biochemical analyses, 20 participants per group were ultimately included, resulting in a total sample size of 60.

All statistical analyses were performed using GraphPad Prism version 10.0 (GraphPad Software, San Diego, CA, USA). The normality of data distribution was assessed using the Shapiro–Wilk test, and homogeneity of variances was evaluated with Levene’s test. Intergroup comparisons were performed using one-way analysis of variance (ANOVA) with Tukey’s post hoc test. Categorical variables were analyzed using the chi-square test.

Confidence intervals were calculated for descriptive statistics to aid interpretation of the estimate’s precision and clinical relevance. 

Because age differed significantly between groups, analysis of covariance (ANCOVA) was performed to adjust group comparisons for age. Homogeneity of regression slopes was evaluated by testing group-by-age interactions before interpreting adjusted group effects.

When interactions were not significant, group differences were assessed using a common slope model.

Additional sensitivity analyses were conducted using separate ANCOVA models, including plaque index (PI) and probing depth (PD) as covariates, to assess the robustness of group comparisons.

When appropriate, Student’s t-test was used for post hoc comparisons. Correlations between variables were determined using Spearman’s correlation analysis. Given the evaluation of multiple biomarkers and multiple correlation analyses, the potential for type I error inflation was considered. Therefore, correlation analyses were interpreted as exploratory, with emphasis on consistency and biological plausibility rather than on isolated statistically significant *p*-values. Formal adjustment for multiple comparisons was not applied in order to avoid excessive type II error in this hypothesis-generating study.

A *p*-value of less than 0.05 (α = 0.05) was considered statistically significant for all tests. 

## 3. Results

### 3.1. Study Population and Periodontal Clinical Parameters

The demographic characteristics and clinical periodontal parameters of the study population are summarized in Table 1. The distribution of gender did not differ significantly among EC users, CS, and NS (*p* = 0.338). However, a significant difference in age was observed between groups (*p* = 0.002), with CS and NS being significantly older than EC users (23.55 ± 3.55, 95% CI: 21.89–25.21; 29.70 ± 6.61, 95% CI: 26.61–32.79; 27.75 ± 5.32, 95% CI: 25.26–30.24, respectively, for EC users, CS, and NS) with a large effect size (η^2^ = 0.198).

Mean PD values were significantly higher in both EC users (1.95 ± 0.34 mm, 95% CI: 1.793–2.114) and CS (1.81 ± 0.40 mm, 95% CI: 1.621–1.997) compared with NSs (1.63 ± 0.33 mm, 95% CI: 1.473–1.784; *p* = 0.022), with a moderate effect size (η^2^ = 0.126). Although PI, CAL, and BOP scores were slightly higher in EC users and CS than in NS, these differences did not reach statistical significance (*p* > 0.05). GCF volume was comparable among the three groups (*p* = 0.748). Smoking-related variables differed between the EC and CS groups. CS exhibited significantly longer smoking duration (4.46 ± 2.07, 95% CI: 3.066–5.398 and 7.19 ± 3.94, 95% CI: 5.090–9.285, respectively, for EC users and CS; *p* = 0.028), with a large effect size (η^2^ = 0.188) and higher daily cigarette consumption (8.91 ± 2.74, 95% CI: 7.070–10.750 and 14.44 ± 6.62, 95% CI: 11.150–17.740, respectively, for EC users and CS; *p* = 0.004), compared with EC users with a large effect size (η^2^ = 0.286).

### 3.2. Biochemical Findings

ROS levels were similar among EC users (503.1 ± 60.49, 95% CI: 487.5–518.7), CS (489.3 ± 87.21, 95% CI: 464.0–514.6), and NS (484.5 ± 58.95, 95% CI: 469.3–499.7) (*p* > 0.05).

In contrast, MMP-9 concentrations were significantly higher in CS than in both EC users (*p* = 0.0283) and NSs (*p* = 0.0070) (72.96 ± 12.89, 95% CI: 69.54–76.38; 81.59 ± 22.62, 95% CI: 75.42–87.76; 69.30 ± 14.38, 95% CI: 63.93–74.67, respectively, for EC users, CS, and NS), with a small effect size (η^2^ = 0.077).

**Figure 2:**
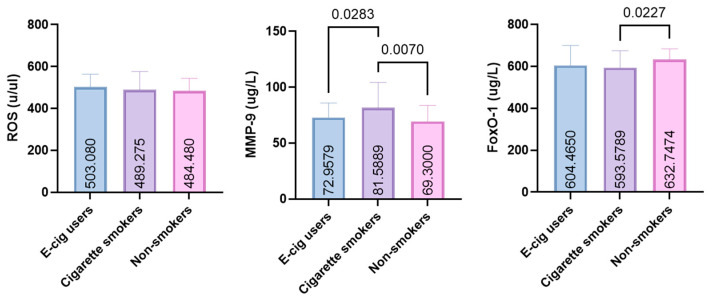
GCF levels of FOXO-1, MMP-9, and ROS in e-cigarette users, smokers, and non-smokers (ROS: reactive oxygen species; MMP-9: matrix metalloproteinase-9; FOXO-1: forkhead box protein O-1).

FOXO-1 levels were significantly lower in CS than in NS (*p* = 0.0227), whereas EC users showed values intermediate between the two groups (604.5 ± 95.45, 95% CI: 579.8–629.1; 593.6 ± 81.00, 95% CI: 572.1–615.1; 632.7 ± 51.06, 95% CI: 619.2–646.3, respectively, for EC users, CS, and NS), with a small effect size (η^2^ = 0.024) (Figure 2).

Therefore, subsequent group comparisons of biochemical outcomes were performed using analysis of covariance (ANCOVA) to account for potential confounding by age. In addition, PI and PD were included as covariates in separate ANCOVA models to evaluate the robustness of group differences with respect to periodontal variables.

As shown in Table 2, no significant group-by-covariate interactions were observed for ROS, MMP-9, or FOXO-1 across models adjusted for age, PI, or PD (all *p* > 0.05), indicating homogeneity of regression slopes and supporting the use of common slope models. After adjustment, ROS levels did not differ significantly between groups in models adjusted for age (*p* = 0.305), PI (*p* = 0.314), or PD (*p* = 0.310).

In contrast, MMP-9 levels remained significantly different between groups after adjustment for age (F (2, 137) = 5.963, *p* = 0.003), PI (F (2, 137) = 5.879, *p* = 0.004), and PD (F (2, 137) = 5.635, *p* = 0.004), indicating that the observed group differences were independent of baseline age and periodontal indices. Similarly, FOXO-1 levels showed significant adjusted group differences after controlling for age (F (2, 170) = 4.170, *p* = 0.017), PI (F (2, 170) = 3.788, *p* = 0.025), and PD (F (2, 170) = 3.748, *p* = 0.026). 

Estimated marginal means derived from these ANCOVA models are presented in Table 3. After adjustment for age, PI, or PD, CS consistently exhibited higher adjusted MMP-9 values compared with EC users and NS, whereas FOXO-1 levels were lowest in CS and highest in NS across all adjusted models. These patterns were consistent across all covariates included, supporting the robustness of the adjusted group comparisons. 

### 3.3. Correlation Analysis

Spearman’s rank correlation analyses were conducted separately within each study group to evaluate associations among oxidative stress-related biomarkers and exposure characteristics (Table 4). In EC users, significant positive correlations were observed among all three biomarkers. ROS levels were strongly correlated with both MMP-9 (r = 0.622, *p* < 0.001) and FOXO-1 (r = 0.604, *p* < 0.001). In addition, a strong positive correlation was detected between MMP-9 and FOXO-1 (r = 0.733, *p* < 0.001). No significant associations were identified between biomarker levels and either the duration of EC use or daily puff frequency.

Among CS, FOXO-1 levels showed a strong positive correlation with ROS (r = 0.650, *p* < 0.001) and MMP-9 (r = 0.662, *p* < 0.001). Daily cigarette consumption was negatively correlated with MMP-9 levels (r = −0.513, *p* < 0.05), whereas no significant correlations were observed between biomarker levels and smoking duration.

In non-smokers, no statistically significant correlations were observed among ROS, MMP-9, and FOXO-1.

## 4. Discussion

This study evaluated oxidative stress-related and tissue-degradative biomarkers in the GCF of periodontally healthy CS, EC users, and NS. Among the biomarkers assessed, MMP-9 and FOXO-1 emerged as particularly informative indicators of tissue response to smoking exposure. CS exhibited significantly higher MMP-9 levels and lower FOXO-1 concentrations than NS, whereas EC users showed intermediate values between the two groups. ROS levels did not differ significantly among groups; however, FOXO-1 showed significant positive correlations with both ROS and MMP-9 in exposed groups, particularly among EC users and CS, indicating concurrent alterations in oxidative stress-related and matrix-regulatory biomarkers within the gingival microenvironment. The clinical periodontal findings further support a cautious interpretation of the observed biochemical alterations. In the present study, PI values were comparable among groups, indicating similar oral hygiene status at the time of examination. Although PD values were modestly higher in EC users and CS than in NS, all measurements remained within ranges consistent with periodontal health. The dissociation between plaque accumulation and PD suggests that these small differences are unlikely to reflect plaque-driven periodontal breakdown. Rather, they may represent subtle, subclinical tissue responses associated with nicotine or aerosol exposure, occurring in the absence of attachment loss or overt inflammatory destruction. Importantly, these statistically significant differences should not be interpreted as evidence of early periodontal disease without longitudinal confirmation. This interpretation is consistent with clinical and optical studies using quantitative light-induced fluorescence, which demonstrate that smokers exhibit the most pronounced oral hygiene and biofilm-related alterations, whereas electronic nicotine delivery system users display intermediate changes that are generally modest and closer to never-smokers [28]. Similarly, population-based studies in young EC users report mild gingival and plaque-related findings in the absence of established periodontal disease [29]. Taken together, preserved plaque control, minimal variation in probing depth, and the absence of attachment loss support the view that the clinical differences observed in this cohort reflect subtle exposure-associated alterations rather than clinically meaningful periodontal pathology.

Age represents a relevant biological factor in periodontal homeostasis [30] and oxidative stress regulation [31], and the observed age differences between exposure groups therefore warrant consideration. In the present study, EC users were younger on average than CS and NS, reflecting broader epidemiologic trends in nicotine product use. To account for the potential confounding influence of age, group comparisons of biochemical outcomes were subsequently evaluated using analysis of covariance with age included as a covariate. Importantly, the observed group differences in MMP-9 and FOXO-1 remained statistically significant after adjustment for age, whereas ROS levels were comparable across groups. These findings suggest that the associations observed between smoking status and matrix-regulatory biomarkers are not solely attributable to baseline age differences. Nevertheless, given the cross-sectional design, residual confounding cannot be entirely excluded, and age-related biological variability may still contribute to interindividual differences in oxidative and inflammatory responses. Accordingly, future longitudinal studies with age-matched cohorts or age-stratified analyses will be essential to further clarify the independent and interactive effects of age and nicotine exposure on periodontal biology.

These findings reinforce the concept that conventional CS promotes inflammation, oxidative imbalance, and enhanced proteolytic activity within periodontal tissues. Elevated MMP-9 levels in CS are consistent with increased extracellular matrix turnover, in line with a previous study reporting higher GCF concentrations of MMP-9 and myeloperoxidase in CS compared with EC users and NS, indicating greater oxidative and enzymatic activity due to tobacco combustion [24]. The observed reduction in FOXO-1 levels among CS may represent suppression of antioxidant and tissue-protective pathways, which could further amplify oxidative injury and impair wound healing capacity.

EC users, while displaying biomarker profiles intermediate between CS and NS, still demonstrated deviations from normal physiological levels, indicating that EC exposure is not biologically inert. These results align with recent evidence that EC aerosols, though devoid of combustion products, contain nicotine and reactive compounds capable of inducing oxidative stress, inflammatory responses, and cellular dysfunction in periodontal tissues [32,33]. Recent meta-analyses have also confirmed that electronic nicotine delivery systems are biologically active and can cause moderate inflammatory and microbial alterations [10]. Notably, EC users demonstrated shorter exposure duration and lower daily use intensity compared with conventional CS; nevertheless, their biomarker interaction profiles showed similarities to those observed in long-term smokers. These similarities suggest overlapping molecular associations rather than equivalent biological effects, particularly given differences in exposure intensity and duration.

The observed reduction in FOXO-1 levels among smokers provides insight into the molecular consequences of oxidative exposure. FOXO-1, a transcription factor with antioxidant and cytoprotective properties, also regulates genes involved in inflammation, apoptosis, and extracellular matrix remodeling [34,35]. Under physiological conditions, FOXO-1 promotes the transcription of antioxidant enzymes such as superoxide dismutase and catalase, thereby maintaining redox homeostasis. However, excessive oxidative stress can modify FOXO-1 through acetylation or phosphorylation, leading to its cytoplasmic translocation and functional inactivation [25,34]. Experimental evidence also indicates that FOXO-1 may directly bind to the MMP-9 promoter and enhance its transcription under oxidative or inflammatory conditions [19]. Thus, the positive correlation between FOXO-1 and MMP-9 observed in exposed groups in this study may not necessarily reflect a purely protective role of FOXO-1, but rather a complex adaptive response to oxidative stress. During early or moderate stress states, FOXO-1 activation may coincide with MMP-9 upregulation as part of a coordinated cellular effort to remodel extracellular matrix components and maintain tissue homeostasis [36,37]. Under chronic or excessive oxidative conditions, however, persistent ROS exposure appears to suppress FOXO-1 activity while sustaining MMP-9 expression, thereby promoting matrix degradation and tissue breakdown [38]. This dual regulatory behavior supports the view that FOXO-1 acts as both a guardian of redox balance and a mediator of stress-induced matrix remodeling, depending on the intensity and duration of oxidative challenge [39]. From a clinical perspective, this molecular interaction may represent an early adaptive mechanism in otherwise healthy periodontal tissues exposed to nicotine, preceding overt inflammatory or structural damage. In this context, the positive correlations observed between FOXO-1 and MMP-9 in exposed groups should be interpreted as reflecting concurrent regulation of these biomarkers rather than coordinated or sequential signaling pathways. As correlation does not imply causality or directionality, these associations cannot be taken as evidence of mechanistic coupling. Instead, they may represent parallel or adaptive cellular responses to shared environmental or oxidative stressors within the gingival microenvironment.

Interestingly, although CS exhibited higher overall MMP-9 levels compared with NS, a negative correlation was observed between daily cigarette consumption and MMP-9 levels within the CS group. Given the modest sample size and the exploratory nature of within-group correlation analyses, this finding should be interpreted with caution and does not permit definitive conclusions regarding a nonlinear or paradoxical biological effect of smoking intensity on MMP-9 regulation.

Accordingly, this observation is unlikely to represent a stable dose–response relationship and is more plausibly attributable to inter-individual variability in host inflammatory responses among smokers. In this context, previous studies have reported that long-term or heavy smokers may exhibit attenuated clinical and biochemical inflammatory responses despite ongoing tissue injury, a phenomenon commonly attributed to immune cell dysfunction or exhaustion [40,41].

Taken together, the present finding should be regarded as hypothesis-generating rather than mechanistically conclusive, highlighting the complexity of inflammatory regulation in smokers and underscoring the need for adequately powered, longitudinal studies specifically designed to evaluate exposure intensity and biomarker dynamics over time.

The absence of significant differences in ROS levels among groups may reflect compensatory antioxidant mechanisms that help maintain redox balance in clinically healthy periodontal sites. However, concurrent changes in FOXO-1 and MMP-9 expression indicate the presence of subclinical biochemical alterations. Previous studies have shown that EC users exhibit elevated pro-inflammatory cytokines and altered metabolomic profiles compared to non-smokers [42,43,44]. This suggests that biochemical changes can precede clinical periodontal inflammation. In addition, previous studies examining gingival crevicular fluid and saliva have indicated that total oxidant and antioxidant capacities may vary with periodontal conditions independently of individual ROS measurements, suggesting that compensatory antioxidant defenses can maintain redox balance even in the face of environmental stressors [45].

Elevated MMP-9 levels in CS reinforce the established role of matrix metalloproteinases in tobacco-induced tissue degradation. MMP-9 contributes to the breakdown of the extracellular matrix, alveolar bone resorption, and loss of connective tissue [46,47]. The present findings confirm that exposure to cigarette smoke activates molecular pathways involved in collagen degradation. This is consistent with previous reports of increased MMP expression and marginal bone loss in smokers [38]. EC users displayed intermediate MMP-9 levels, indicating a biological effect less severe than CS but distinct from NS. These results demonstrate that EC use also disrupts periodontal homeostasis. 

A key strength of this study is the exclusive inclusion of periodontally healthy individuals. This allows for a controlled assessment of the effects of nicotine and aerosol compounds on gingival biochemistry. This approach minimizes confounding from pre-existing inflammation and isolates the early biochemical impact of nicotine. The findings suggest that oxidative and matrix-degrading responses can occur before visible clinical signs of periodontal destruction become apparent. These early molecular changes suggest that exposure to CS and EC aerosols predisposes periodontal tissues to a pro-oxidative, catabolic state. This may raise the risk of inflammatory breakdown when other risk factors are present.

Beyond the biochemical context, the demographic differences observed in our study, particularly the younger age of EC users, are consistent with global epidemiologic data. The World Health Organization (WHO) recently reported that adolescents and young adults use EC at higher rates than adults in all WHO regions, emphasizing the rapid uptake of these devices among younger populations [48]. This phenomenon has been attributed to their wide availability, diverse flavor options, discreet design, and the misleading perception that ECs are a safer alternative to traditional smoking [48]. Early exposure to these aerosolized nicotine products may lead to the initiation of addictive behavior and to subtle biological alterations even in the absence of clinical periodontal inflammation. Collectively, these findings underscore the importance of public health strategies aimed at restricting youth access to ECs and increasing awareness of their potential oral and systemic effects. Although broader epidemiological data indicate rising EC use among adolescents and young adults, these observations do not address the internal validity limitations of the present study. Accordingly, these public health data are cited solely to contextualize the relevance of investigating subclinical biomarker variations in otherwise healthy periodontal tissues.

Several limitations of this study should be acknowledged. The cross-sectional design precludes causal inference regarding the relationship between nicotine exposure and biomarker alterations. Measurements of ROS and enzymatic activity in GCF represent a single time-point assessment and were obtained using an ELISA-based method that detects stable ROS-related oxidative products rather than free-radical species. Accordingly, the measured ROS values provide an indirect estimate of local oxidative burden rather than a direct measure of real-time oxidative activity; however, this approach is commonly used to enable standardized, comparative assessment of oxidative stress across study groups. In addition, exposure assessment was based primarily on self-reported smoking and EC use, and not all reports were biochemically verified. This may introduce exposure misclassification, particularly with respect to exclusive EC use and NS status. Although strict inclusion and exclusion criteria were applied to minimize misclassification, the possibility of undetected dual use or recent smoking cannot be fully excluded. Such misclassification would be expected to bias group differences toward the null, suggesting that the observed associations, particularly for MMP-9, may represent conservative estimates of exposure-related differences. Another limitation of the present study is the relatively limited characterization of EC exposure. Exposure assessment was based on self-reported duration of use and average daily puff frequency, without detailed information on device generation, power or temperature settings, nicotine concentration, or flavor composition. Because aerosol composition, toxicant delivery, and nicotine exposure can vary substantially depending on device characteristics and user behavior, the findings should be interpreted as reflecting general associations with EC exposure rather than device-specific or dose-dependent biological effects. Another consideration is the distribution of sex across study groups. Although the difference in sex distribution did not reach statistical significance, the NS group included a higher proportion of males, whereas the EC user group was predominantly female. Given established sex-related differences in immune regulation, oxidative stress responses, and periodontal tissue metabolism, sex may have influenced biomarker expression. However, the relatively small sample size limited the statistical power to conduct stratified or sex-adjusted analyses. Future studies with larger cohorts should consider sex-stratified or multivariable models to more fully evaluate potential sex-specific effects. Although the use of a single calibrated examiner minimizes inter-examiner variability, it may limit generalizability, and future studies would benefit from multi-examiner designs with inter-examiner reliability assessment. Finally, because multiple biomarkers and correlation analyses were evaluated in a relatively small sample, the observed associations should be interpreted cautiously and considered exploratory pending confirmation in larger, adequately powered studies. Despite these limitations, the present study provides valuable baseline data on early biochemical alterations associated with nicotine and aerosol exposure in periodontally healthy individuals.

## 5. Conclusions

In conclusion, this cross-sectional study identified associations between CS and EC use and variations in oxidative stress- and matrix-related biomarkers in the gingival crevicular fluid of periodontally healthy individuals. Differences in MMP-9 and FOXO-1 levels were observed across exposure groups in the absence of overt periodontal disease, suggesting the presence of subclinical biochemical variability within clinically healthy periodontal tissues. Given the observational design and reliance on self-reported exposure, these findings should be interpreted as descriptive and hypothesis-generating rather than definitive evidence of biological impact or temporal progression. From a clinical and public health perspective, the results underscore the importance of continued surveillance and prevention-oriented strategies addressing both CS and EC use. Longitudinal studies with refined exposure assessment and robust multivariable modeling are required to elucidate whether the observed biomarker patterns precede clinically meaningful periodontal deterioration or instead represent stable subclinical adaptations within otherwise healthy tissues.

## Figures and Tables

**Table 1:** Comparison of Clinical Periodontal Parameters Between Groups.

Variables	E-Cig Users	Cigarette Smokers	Non-Smokers	*p*-Value	Effect Size (η^2^)
	(EC, *n* = 20)	(CS, *n* = 20)	(NS, *n* = 20)		
Gender (M/F)	7/13	9/11	14/6	0.338	-
Age (year)	23.55 ± 3.55	29.70 ± 6.61 *	27.75 ± 5.32 *	**0.002**	0.198
	(21.89–25.21)	(26.61–32.79)	(25.26–30.24)		
PI	0.995 ± 0.501	0.926 ± 0.567	0.751 ± 0.460	0.206	0.054
	(0.760–1.229)	(0.661–1.191)	(0.450–0.930)		
Smoking duration (years)	4.455 ± 2.067	7.188 ± 3.936 *	-	**0.028**	0.188
	(3.066–5.398)	(5.090–9.285)			
Number of cigarettes (n/day)	8.909 ± 2.737	14.440 ± 6.617 *	-	**0.004**	0.286
	(7.070–10.750)	(11.15–17.74)			
PD (mm)	1.954 ± 0.342	1.809 ± 0.402	1.629 ± 0.333 *	**0.022**	0.126
	(1.793–2.114)	(1.621–1.997)	(1.473–1.784)		
BOP (%)	7.884 ± 2.193	7.220 ± 2.834	7.630 ± 2.083	0.678	0.014
	(6.858–8.910)	(5.894–8.546)	(6.655–8.605)		
CAL (mm)	1.090 ± 0.986	0.739 ± 0.732	0.995 ± 0.887	0.427	0.029
	(0.628–1.551)	(0.396–1.081)	(0.580–1.411)		
GCF volume (μL)	46.21 ± 26.65	40.57 ± 29.24	39.77 ± 18.43	0.748	0.115
	(30.10–62.31)	(26.88–54.25)	(31.14–48.39)		

PI: plaque index; PD: probing depth; BOP: bleeding on probing; CAL: clinical attachment level; GCF volume: gingival crevicular fluid volume. Values are presented as mean ± standard deviation (SD); values in parentheses indicate 95% confidence intervals (CI). Statistical analysis performed using one-way ANOVA. Bolded values indicate statistically significant differences (*p* < 0.05). * Statistically significantly different between the EC group.

**Table 2:** Age- and periodontal-adjusted group comparisons of FOXO-1, ROS, and MMP-9.

Variable		Group–Variable Interaction		Group Effect Adjusted for Variable	
		F (df)	*p* Value	F (df)	*p* Value
ROS	Age	1.031 (2, 162)	0.359	1.197 (2, 164)	0.305
	PI	0.397 (2, 162)	0.673	1.167 (2, 164)	0.314
	PD	0.439 (2, 162)	0.645	1.181 (2, 164)	0.310
MMP-9	Age	0.094 (2, 135)	0.910	5.963 (2, 137)	**0.003**
	PI	0.209 (2, 135)	0.812	5.879 (2, 137)	**0.004**
	PD	1.369 (2, 135)	0.258	5.635 (2, 137)	**0.004**
FOXO-1	Age	0.270 (2, 168)	0.764	4.170 (2, 170)	**0.017**
	PI	0.404 (2, 168)	0.668	3.788 (2, 170)	**0.025**
	PD	1.956 (2, 168)	0.145	3.748 (2, 170)	**0.026**

ROS: reactive oxygen species; MMP-9: matrix metalloproteinase-9; FOXO-1: forkhead box protein O-1, PI: plaque index; PD: probing depth. Analysis of covariance (ANCOVA) was used to compare e-cigarette users, cigarette smokers, and non-smokers with age, PI, or PD included as covariates in separate models. Homogeneity of regression slopes was assessed by testing group–covariate interactions. Adjusted group effects were evaluated using a common-slope model when interactions were not significant. Bolded values indicate statistically significant differences (*p* < 0.05).

**Table 3:** Estimated marginal means of MMP-9 and FOXO-1 adjusted for age and periodontal variables.

Variables		E-Cig Users	Cigarette Smokers	Non-Smokers
		(EC, *n* = 20)	(CS, *n* = 20)	(NS, *n* = 20)
MMP-9	Adjusted for age	72.81	81.54	68.71
	Adjusted for PI	72.98	81.71	69.06
	Adjusted for PD	72.91	81.22	69.35
FOXO-1	Adjusted for age	604.5	592.9	633.1
	Adjusted for PI	604.5	593.6	632.7
	Adjusted for PD	604.5	593.9	632.9

MMP-9: matrix metalloproteinase-9; FOXO-1: forkhead box protein O-1, PI: plaque index; PD: probing depth. Estimated marginal means were derived from separate ANCOVA models with age, PI, or PD included as covariates in separate models.

**Table 4:** Correlation analyses of biomarkers and exposure characteristics by study group.

E-Cigarette Users (*n* = 20)			
Variables	ROS	MMP-9	FOXO-1
ROS		0.622 **	0.604 **
MMP-9			0.733 **
Duration of e-cigarette use (years)	−0.268	−0.367	−0.249
Daily e-cigarette use (puffs/day)	0.018	−0.251	−0.110
Cigarette smokers (*n* = 20)			
Variables	ROS	MMP-9	FOXO-1
ROS		0.441	0.650 **
MMP-9			0.662 **
Duration of smoking (years)	−0.167	−0.165	−0.219
Number of cigarettes (n/day)	−0.068	−0.513 *	−0.162
Non-smokers (*n* = 20)			
Variables	ROS	MMP-9	FOXO-1
ROS		−0.162	0.412
MMP-9			0.399

ROS: reactive oxygen species; MMP-9: matrix metalloproteinase-9; FOXO-1: forkhead box protein O-1. Spearman’s rank correlation analyses were conducted within each group.* *p* < 0.05; ** *p* < 0.001.

## Data Availability

The data supporting this study’s findings are available from the corresponding author upon reasonable request.

## Abbreviations

The following abbreviations are used in this manuscript:

GCF	Gingival Crevicular Fluid
ROS	Reactive Oxygen Species
MMP-9	Matrix Metalloproteinase-9
FOXO-1	Forkhead Box Protein O-1
EC	Electronic cigarette
CS	Cigarette Smoker
NS	Non-Smoker
PD	Probing Depth
CAL	Clinical Attachment Level
BOP	Bleeding on Probing
PI	Plaque Index
WHO	World Health Organization
